# Characterization of the Metabolically Modified Heavy Metal-Resistant *Cupriavidus metallidurans* Strain MSR33 Generated for Mercury Bioremediation

**DOI:** 10.1371/journal.pone.0017555

**Published:** 2011-03-14

**Authors:** Luis A. Rojas, Carolina Yáñez, Myriam González, Soledad Lobos, Kornelia Smalla, Michael Seeger

**Affiliations:** 1 Laboratorio de Microbiología Molecular y Biotecnología Ambiental, Departamento de Química and Center for Nanotechnology and Systems Biology, Universidad Técnica Federico Santa María, Valparaíso, Chile; 2 Laboratorio de Espectroscopía, Facultad de Farmacia, Universidad de Valparaíso, Playa Ancha, Valparaíso, Chile; 3 Julius Kühn-Institut, Federal Research Centre for Cultivated Plants (JKI), Institute for Epidemiology and Pathogen Diagnostics, Braunschweig, Germany; University of Kansas, United States of America

## Abstract

**Background:**

Mercury-polluted environments are often contaminated with other heavy metals. Therefore, bacteria with resistance to several heavy metals may be useful for bioremediation. *Cupriavidus metallidurans* CH34 is a model heavy metal-resistant bacterium, but possesses a low resistance to mercury compounds.

**Methodology/Principal Findings:**

To improve inorganic and organic mercury resistance of strain CH34, the IncP-1β plasmid pTP6 that provides novel *merB*, *merG* genes and additional other *mer* genes was introduced into the bacterium by biparental mating. The transconjugant *Cupriavidus metallidurans* strain MSR33 was genetically and biochemically characterized. Strain MSR33 maintained stably the plasmid pTP6 over 70 generations under non-selective conditions. The organomercurial lyase protein MerB and the mercuric reductase MerA of strain MSR33 were synthesized in presence of Hg^2+^. The minimum inhibitory concentrations (mM) for strain MSR33 were: Hg^2+^, 0.12 and CH_3_Hg^+^, 0.08. The addition of Hg^2+^ (0.04 mM) at exponential phase had not an effect on the growth rate of strain MSR33. In contrast, after Hg^2+^ addition at exponential phase the parental strain CH34 showed an immediate cessation of cell growth. During exposure to Hg^2+^ no effects in the morphology of MSR33 cells were observed, whereas CH34 cells exposed to Hg^2+^ showed a fuzzy outer membrane. Bioremediation with strain MSR33 of two mercury-contaminated aqueous solutions was evaluated. Hg^2+^ (0.10 and 0.15 mM) was completely volatilized by strain MSR33 from the polluted waters in presence of thioglycolate (5 mM) after 2 h.

**Conclusions/Significance:**

A broad-spectrum mercury-resistant strain MSR33 was generated by incorporation of plasmid pTP6 that was directly isolated from the environment into *C. metallidurans* CH34. Strain MSR33 is capable to remove mercury from polluted waters. This is the first study to use an IncP-1β plasmid directly isolated from the environment, to generate a novel and stable bacterial strain useful for mercury bioremediation.

## Introduction

Environmental decontamination of polluted sites is one of the main challenges for sustainable development. Bioremediation is an attractive technology for the clean-up of polluted waters and soils [1]–[6]. Mercury is one of the most toxic elements in the environment [7], [8]. Metal mining, fossil combustion and the chloralkali and acetaldehyde industries have raised mercury levels in water bodies and soils. Mercury enters from industrial sources mainly as Hg^2+^ into the environment [9]–[11]. Physicochemical and biological processes have been applied for mercury removal from contaminated environments. Physicochemical processes for heavy metal removal such as ion exchange and precipitation treatment procedures result in large volumes of mercury-contaminated sludge and are of high cost [12]–[13]. As an alternative to physicochemical processes, bacteria have been applied for the remediation of mercury pollution [1], [3], [10], [14]. The biological processes for mercury removal are of low cost, simple and environmentally friendly [10]. Mercury-polluted sites are often contaminated with other heavy metals [15]. Therefore, bacteria with resistance to several heavy metals may be useful for remediation.

**Figure 1:**
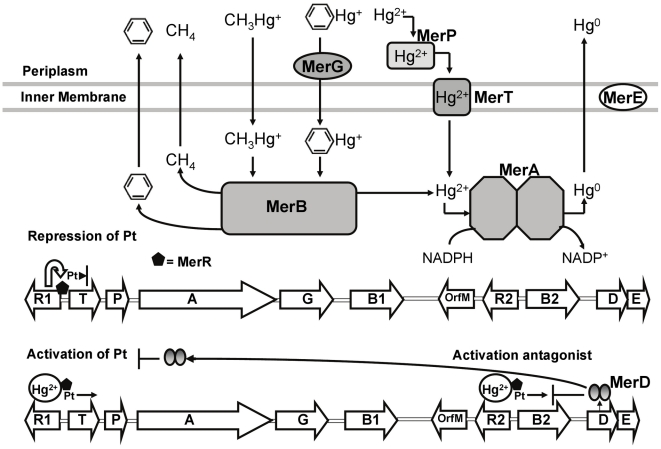
Bacterial mechanisms for organic and inorganic mercury detoxification and regulation of the *mer* genes. MerA, mercuric reductase; MerB, organomercurial lyase; MerP, periplasmic mercury-binding protein; MerT, membrane mercury transport protein; MerG, periplasmic protein involved in cell permeability to phenylmercury; MerE membrane protein that probably acts as a broad mercury transporter; MerR, transcriptional activator or repressor of the transcription of *mer* genes (black pentagon); MerD, co-represor of transcriptional activation; Pt, promoter of *merT* and *merB2* genes.

In bacteria, mercury resistance *mer* genes are generally organized in operons located on plasmids and transposons [16]–[17]. The narrow-spectrum mercury resistance *merRTPADE* operon confers resistance to inorganic mercury and the broad-spectrum mercury resistance *merRTPAGBDE* operon confers resistance to inorganic and organic mercury species (Fig. 1). The bacterial mechanism of mercury resistance includes the uptake and transport of Hg^2+^ by the periplasmic protein MerP and the inner membrane protein MerT. MerE is a membrane protein that probably acts as a broad mercury transporter mediating the transport of both methylmercury and Hg^2+^ [18]. The cytosolic mercuric reductase MerA reduces Hg^2+^ to less toxic elemental mercury [9]. The gene *merB* encodes an organomercurial lyase that catalyses the protonolytic cleavage of carbon-mercury bonds [19]–[20]. The *merG* gene product is involved in the reduction of cellular permeability to organomercurial compounds [21]. MerD probably acts as a distal co-repressor of transcriptional activation [9], [22]. MerR is the activator or repressor of the transcription of *mer* genes in presence or absence of mercury ions, respectively [23], [24]. At mercury stress condition the transcriptional activator MerR triggers the expression of the structural *mer* genes [25]. Sequencing of the native IncP-1β plasmid pTP6 that was originally isolated from mercury-polluted river sediment in a triparental mating showed that all these genes are part of transposon Tn*50580* [26].

The heavy metal-resistant model bacterium *Cupriavidus metallidurans* strain CH34 harbors two large plasmids, pMOL28 and pMOL30, which carry genetic determinants for heavy metal resistance [27]. Each plasmid contains a *merRTPADE* operon that confers a narrow-spectrum mercury resistance. To improve inorganic and organic mercury resistance of strain *C. metallidurans* CH34, the IncP-1β plasmid pTP6 was introduced into strain CH34 in this study. The transconjugant strain *Cupriavidus metallidurans* MSR33 showed a broad-spectrum mercury resistance and was able to efficiently remove mercury from polluted water.

## Materials and Methods

### Chemicals

HgCl_2_ (analytical grade), CuSO_4_·5H_2_O, K_2_CrO_4_, NaBH_4_, NaOH, HCl (Suprapur) and standard Titrisol solution were obtained from Merck (Darmstadt, Germany). CH_3_HgCl (analytical grade) were obtained from Sigma Aldrich (Saint Louis, MO, USA). Stock solutions of Cu^2+^ (5,000 µg ml^−1^); CrO_4_^2−^ (2,500 µg ml^−1^); Hg^2+^ (1,000 µg ml^−1^) and CH_3_Hg^+^ (100 µg ml^−1^) were prepared. NaBH_4_ solution (0.25%) was prepared in NaOH (0.4%) solution. High purity hydrochloric acid was used for mercury dilutions before quantification by inductively coupled plasma optical emission spectrometer (ICP-OES). Sodium succinate and salts for media preparation were obtained from Merck (Darmstadt, Germany). Taq DNA polymerase and bovine serum albumin for PCR were obtained from Invitrogen (Carlsbad, CA, USA). RNA was extracted using an RNeasy Protect Bacteria Mini kit from Qiagen (Hilden, Germany). For RNA quantification the Quant-iT™ RNA Assay kit from Invitrogen (Carlsbad, CA, USA) was used. RT-PCR was performed using SuperScriptTM III One-Step RT-PCR System and Taq DNA Polymerase Platinum® from Invitrogen (Carlsbad, CA, USA).

### Bacteria, plasmids, and culture conditions

*Cupriavidus metallidurans* CH34 [28], [29], *Escherichia coli* JM109 (pTP6) [26] and *Cupriavidus necator* JMP134 (pJP4) [30] were grown in Luria Bertani (LB) medium (contained per l: 10 g tryptone, 5 g yeast extract and 10 g NaCl) [31] in absence or presence of Hg^2+^. *Cupriavidus metallidurans* strains CH34 and MSR33 were cultivated also in low-phosphate Tris-buffered mineral salts (LPTMS) medium. The LPTMS medium contained (per l): 6.06 g Tris, 4.68 g NaCl, 1.49 g KCI, 1.07 g NH_4_Cl, 0.43 g Na_2_SO_4_, 0.2 g MgCl_2_·6H_2_0, 0.03 g CaC1_2_·2H_2_0, 0.23 g Na_2_HPO_4_·12H_2_0, 0.005 g Fe(III)(NH_4_) citrate, and 1 ml of the trace element solution SL 7 of Biebl and Pfennig [28]. Succinate (0.3%) was used as sole carbon source. To study the effect of inorganic mercury on growth, Hg^2+^ (0.04 mM) was added at time 0 or at exponential phase. Bacterial growth was determined by measuring turbidity at 600 nm.

The IncP-1β plasmid pTP6 that was originally isolated in a triparental mating from mercury-polluted river sediments contains *merR1TPAGB1* and *merR2B2D2E* gene clusters conferring a broad-range mercury resistance [26].

### Generation of transconjugant bacterial strains

Plasmid pTP6 was transferred from *E. coli* JM109 to *C. metallidurans* CH34 by biparental mating. Donor and recipient cells were mixed (1∶4) and placed in a sterile filter onto plate count agar (PCA) (Merck, Darmstadt, Germany). After overnight incubation at 28°C, transconjugants were selected on LPTMS agar plates containing succinate (0.3%) as sole carbon source and supplemented with Hg^2+^ (0.04 mM). The transconjugants were further tested for growth in liquid LPTMS medium containing succinate (0.3%) as sole carbon source and supplemented with Hg^2+^ (0.09 mM).

Genomic DNA purification was performed by standard procedures [31]. Plasmid extraction from *C. metallidurans* transconjugants, *C. metallidurans* CH34, *C. necator* JMP134 (pJP4) and *E. coli* JM109 (pTP6) was performed using the method of Kado and Liu with minor modifications [32], [33]. The presence of heavy metal resistance genes in genomic and plasmid DNA was analyzed by polymerase chain reaction (PCR) with specific primers. Primers used for *merB* gene amplification were the forward 5′-TCGCCCCATATATTTTAGAAC-3′ and the reverse 5′-GTCGGGACAGATGCAAAGAAA-3′ [34]. Primers used for amplification of *chrB* gene were the forward 5′-GTCGTTAGCTTGCCAACATC-3′ and the reverse 5′-CGGAAAGCAAGATGTCGAATCG-3′ [35], [36]. Primers used for *copA* gene amplification were the degenerated forward Coprun F2, 5′-GGSASBTACTGGTRBCAC-3′ and the degenerated reverse Coprun R1, 5′-TGNGHCATCATSGTRTCRTT-3′ [37]. Plasmids and PCR products were visualized in agarose gel electrophoresis (0.7%) in TAE buffer (0.04 M Tris, 0.04 M acetate, 0.001 M EDTA, pH 8.0).

The stability of the pTP6 plasmid in strain *C. metallidurans* MSR33 was evaluated through repeated cultivation in LB medium under non-selective conditions. A single colony of *C. metallidurans* MSR33 was used to inoculate 25 ml LB broth. After growth during 24 h at 28°C, serial dilutions were plated on PCA and 25 µl was used to inoculate fresh LB broth. The procedure was repeated six times. Forty-eight colonies randomly selected were picked from PCA plates of appropriate dilutions obtained when grown in LB broth for 1, 2, 3, 4, 5, 6 and 7 d and streaked on PCA supplemented with Hg^2+^ (0.5 mM). Plasmids were isolated and analyzed from 5 randomly selected mercury-resistant colonies from cultures grown during more than 70 generations under non-selective conditions.

### Sequence analyses of MerA and MerB

The amino acid sequences of MerA and MerB proteins were obtained from GenPept database (http://www.ncbi.nlm.nih.gov). The multiple MerA and MerB sequences were aligned using Clustal W software [38].

### Determination of minimum inhibitory concentration (MIC) values

For testing the bacterial resistance to Hg^2+^, CH_3_Hg^+^, Cu^2+^ and CrO_4_^2−^, LPTMS agar plates were used. Ten µl of cultures grown overnight in LPTMS were placed onto the agar plates supplemented with different concentrations of each heavy metal. LPTMS was supplemented with mercury (II) (chloride) in the concentration range from 0.01 to 0.16 mM (in increasing concentration of 0.01 mM steps), copper (sulphate) in the concentration range from 3.0 to 4.2 mM (in increasing concentration of 0.1 mM steps), and (potassium) chromate in the concentration range from 0.5 to 1.0 mM (in increasing concentration of 0.1 mM steps). The plates were incubated at 28°C for 5 d. The lowest concentration of metal salts that prevented growth was recorded as the MIC. MIC for methylmercury in the concentration range from 0.005 to 0.1 mM (in increasing concentration of 0.005 mM steps) was studied on paper discs on plates. The methylmercury in solution (10 µl) was added to sterile paper discs (diameter 6 mm) and the impregnated paper discs were placed on the bacterial culture in an agar plate. Methylmercury diffused in the area surrounding each paper disc. MIC for methylmercury was recorded as the lowest concentration at which growth inhibition was observed. MIC analyses were done in triplicate.

### Transmission electron microscopy

To evaluate the morphology of cells exposed to Hg^2+^, cells were observed by transmission electron microscopy. Strain MSR33 and strain CH34 cells were grown in LPTMS medium and further incubated at exponential phase in presence of Hg^2+^ (0.04 mM) for 2 h. Cells were harvested by centrifugation and washed with 50 mM phosphate buffer (pH 7.0). Cells were fixed and treated for transmission electron microscopy as described [39]. Briefly, the cells were fixed with Karnowsky solution (2.5% glutaraldehyde, 3.0% formaldehyde) in 0.2 M cacodylate buffer, post fixed with 2% osmium tetroxide and dehydrated in ethanol and acetone series. Finally, cells were embedded in an epoxy resin (Eponate 812). Thin sections (500 nm) were obtained with diamond knife in an Ultracut E ultramicrotome (Reichert). Sections were contrasted with uranyl acetate and lead citrate and observed with a Zeiss EM900 electron microscope. Micrographs were obtained and enlarged for analysis.

### Sodium dodecyl sulfate-polyacrylamide gel electrophoresis (SDS-PAGE)

SDS-PAGE was performed as previously reported [40]. Cells grown in LB medium supplemented with Hg^2+^ (0.075 mM) were harvested at exponential phase (Turbidity_600__nm_ = 0.7) and washed twice with 50 mM phosphate buffer (pH 7.0). Cells were treated as previously described [40]. Briefly, Laemmli sample buffer was added (100 µl per 5 mg of wet weight of bacteria), cells were disrupted by boiling for 5 min and cell debris were removed by centrifugation at 10,733 *g* for 10 min at 4°C. Proteins were quantified using a Qubit fluorometer (Invitrogen). Proteins were separated by SDS-PAGE using 7 to 15% linear polyacrylamide gradient and visualized by staining with Coomassie brilliant blue R-250.

### Bioremediation experiments in bioreactors and mercury quantification

A bioreactor was designed for mercury bioremediation assays. Each bioreactor contained 50 ml of Hg^2+^ (0.10 mM and 0.15 mM) spiked aqueous solution (50 mM phosphate buffer, pH 7.0) in a 250 ml sterile flask and was aerated by a blower at 300 ml min^−1^. Volatilized mercury (Hg^0^) was trapped on an external HNO_3_ (10%) solution in a 15 ml sterile tube. Bioreactors were bioaugmented by inoculating MSR33 or CH34 cells grown in LPTMS medium supplemented with Hg^2+^ (0.01 mM) for the induction of *mer* genes (cells were added to reach a final concentration of 2×10^8^ cells ml^−1^). In control bioreactors, cells were not added. Thioglycolate (5 mM) was added to selected bioreactors. The bioreactors were incubated at 30°C for 5 h. All treatments were performed in triplicate. Samples (1 ml) were taken and centrifuged (10,733 *g* for 10 min) to remove the cells. The supernatants were used for mercury quantification. To quantify total mercury, samples were oxidized by adding 5 ml of HNO_3_ (analytical grade), heated at 90°C and then diluted to a final volume of 10 ml with HCl 10% (v/v). Samples were diluted to concentrations <100 µg of Hg per liter. Total mercury was quantified by cold vapor emission spectroscopy using a flow injection system Perkin Elmer (model FIAS 200) linked to an inductively coupled plasma optical emission spectrometer (ICP-OES) Perkin Elmer (model Optima 2000). Ionic mercury (II) from samples was reduced in the FIAS system (1.5 ml min^−1^) with NaBH_4_ (0.25% m/v) in NaOH (0.4% m/v) (1.4 ml min^−1^) to metallic mercury, which was volatilized using argon that carried atomic mercury to the plasma and detected at 253.652 nm. The standard Hg (Titrisol, Merck) concentration in the calibration solutions ranged from 10 to 80 µg l^−1^. Values were calculated as the mean ±SD of three independent experiments.

## Results

### Generation of a broad range mercury-resistant *C. metallidurans* strain

To generate a heavy metal-resistant strain with broad-range mercury species resistance, novel and additional *mer* genes were introduced into heavy metal-resistant *C. metallidurans* CH34. Plasmid pTP6 carrying a complex set of *mer* genes encoding a broad-spectrum mercury resistance was selected to transfer *mer* genes into strain CH34. Plasmid pTP6 is an IncP-1β plasmid that only carries mercury resistance genes including *merB* and *merA* genes and no additional resistance or catabolic genes [26]. MerB1 of pTP6 is closely related to MerB1 of Tn*5058* (*Pseudomonas* sp. strain ED23–33) and MerB from pMR62 (*Pseudomonas* sp. strain K-62), whereas MerB2 of pTP6 is closely to MerB2 of Tn*5058* and MerB of the isolated plasmid pQBR103. MerA from pTP6 is closely related to MerA of Tn*5058* (*Pseudomonas* sp. strain ED23–33) and pMR26 (*Pseudomonas* sp. strain K-62), whereas MerA of the plasmids pMOL28 and pMOL30 are closely related to MerA of the catabolic plasmid pJP4 (*C. necator* strain JMP134) and the virulence plasmid pWR501 (*S. flexneri* strain 5a), respectively.

Plasmid pTP6 was transferred from *E. coli* JM109 (pTP6) to *C. metallidurans* strain CH34 by biparental conjugation. Transconjugants were selected by growth in LPTMS agar medium in presence of Hg^2+^ (0.04 mM). More than 100 mercury-resistant transconjugants were observed in the plate. Parental strain CH34 and *E. coli* strain JM109 (pTP6) did not grow in presence of Hg^2+^ (0.04 mM). All forty-four transconjugants that formed a large colony in presence of Hg^2+^ (0.04 mM) and that were picked were capable to grow in liquid LPTMS medium containing succinate (0.3%) as sole carbon source and Hg^2+^ (0.09 mM). One of these transconjugants, *C. metallidurans* strain MSR33, was selected for further characterization.

### Genetic characterization of the transconjugant strain MSR33

**Figure 2:**
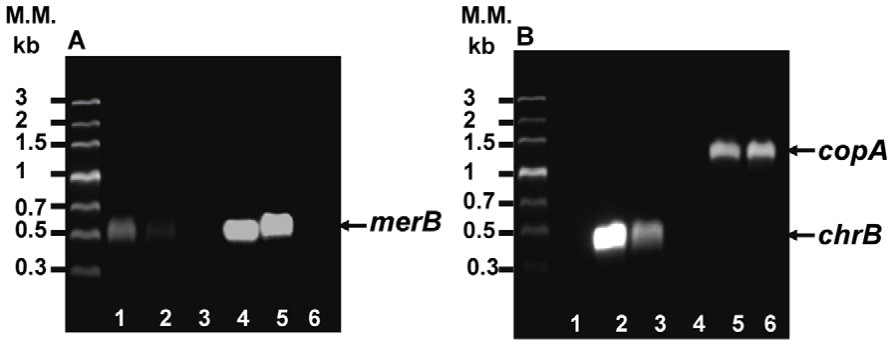
Detection by PCR of heavy metal resistance genes in *C. metallidurans* MSR33. **A**, detection of *merB* gene in plasmid (lanes 1–3) and genomic DNA (lanes 4–6). **B**, detection of *chrB* (lanes 1–3) and *copA* (lanes 4–6) genes in genomic DNA. Lanes: 1 and 4, *E. coli* JM109 (pTP6); 2 and 5, *C. metallidurans* strain MSR33; 3 and 6, *C. metallidurans* strain CH34.

Strain MSR33 was genetically characterized. The presence of plasmid pTP6 in strain MSR33 was confirmed by plasmid isolation and visualization by agarose gel electrophoresis. The presence of *merB* gene in the isolated plasmid, pTP6, and in genomic DNA of strain MSR33 was confirmed by PCR (Fig. 2). Additionally, the *C. metallidurans* chromate-resistance *chrB* gene and the copper resistance *copA* gene were detected in the genome of transconjugant strain MSR33.

The genetic stability of a modified microorganism is critical to its release into the environment [6]. Therefore, the stability of the plasmid pTP6 in the transconjugant strain MSR33 was studied under non-selective conditions. All colonies of strain MSR33 grown under non-selective pressure after 70 generations, maintained their mercury resistance. All the colonies analyzed with improved mercury resistance after 70 generations, possessed the plasmid pTP6. These results indicated that additional mercury resistance *mer* genes were stable in strain MSR33. It has been reported that the IncP-1β plasmid pTP6 was stably maintained in different proteobacterial hosts under no-selective conditions [26].

### Synthesis of MerB and MerA in *C. metallidurans* MSR33

**Figure 3:**
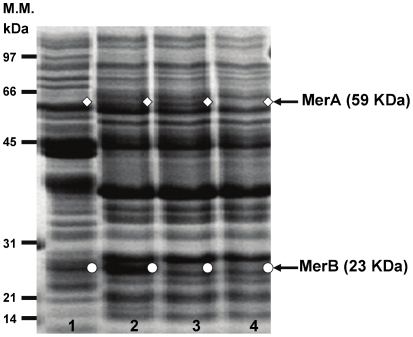
Synthesis of MerB and MerA proteins in *C. metallidurans* strain MSR33. Proteins of cells grown in LPTMS medium in presence (lanes 1–3) and absence of Hg^2+^ (lane 4) were separated by SDS-PAGE and stained with Coomassie blue. Lanes: 1, *E. coli* JM109 (pTP6) grown in presence of Hg^2+^; 2, *C. metallidurans* strain MSR33 grown in presence of Hg^2+^; 3, *C. metallidurans* strain CH34 grown in presence of Hg^2+^; 4, *C. metallidurans* strain CH34 grown in absence of mercury. White diamonds and circles indicate MerA and MerB proteins, respectively. Molecular mass of protein standards in kD are shown on the left side of gel.

Plasmid pTP6 provided to the strain MSR33 novel genes that encode two organomercurial lyases MerB and an additional gene that encodes the mercuric reductase MerA. The synthesis of MerB and MerA in strain MSR33 grown in presence of Hg^2+^ was studied. The protein pattern of strain MSR33 in presence of Hg^2+^ was analyzed by SDS-PAGE (Fig. 3). The induction of proteins of approximately 23 kDa that probably are MerB enzymes was observed only in the transconjugant strain MSR33. Additionally, the induction of a protein of 50 KDa that probably is the mercuric reductase MerA was observed in strains MSR33 and CH34 during growth in presence of Hg^2+^.

### Heavy metal resistance of strain MSR33

In order to study the inorganic mercury and organic mercury resistances of strain MSR33, cells were grown on LPTMS agar plates in presence of Hg^2+^ and CH_3_Hg^+^. Strain MSR33 showed a high resistance to Hg^2+^ (0.12 mM) whereas strain CH34 has a lower resistance to Hg^2+^ (0.05 mM). Therefore, the presence of plasmid pTP6 increased 2.4 fold Hg^2+^ resistance of strain MSR33. Noteworthy, strain MSR33 possesses a high resistance to methylmercury (0.08 mM). In contrast, strain CH34 was sensitive to methylmercury. Strain MSR33 maintained the Cu^2+^ and CrO_4_^2−^ resistances of the parental strain CH34 (Table 1).

**Table 1:** Minimum inhibitory concentrations of heavy metals for transconjugant *C. metallidurans* strain MSR33.

Metal (mM)	Strain MSR33	Strain CH34	Increased resistance (fold)
Hg^2+^	0.12	0.05	2.4
CH_3_Hg^+^	0.08	<0.005	>16
Cu^2+^	3.80	3.80	0
CrO_4_^2−^	0.70	0.70	0

### Effect of Hg^2+^ on the growth and cell morphology

**Figure 4:**
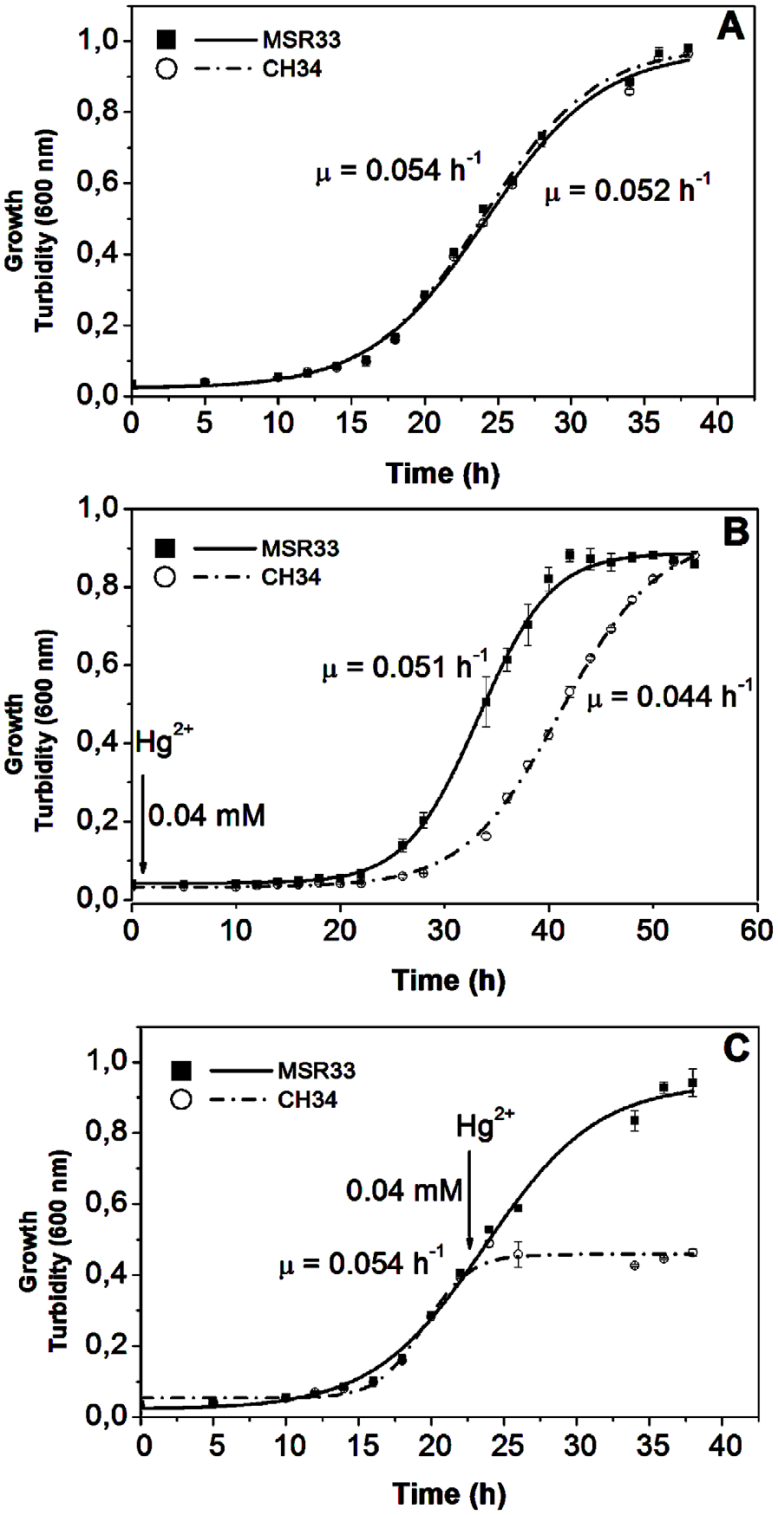
Effect of Hg^2+^ on the growth of *C. metallidurans* strains MSR33 and CH34. Cells were grown in LPTMS medium in absence of Hg^2+^ **A**, presence of Hg^2+^ (0.04 mM) **B**, or Hg^2+^ (0.04 mM) were added to LPTMS medium at exponential phase **C**. The exposure to Hg^2+^ started at the point indicated with an arrow. Each value is the mean ± SD of three independent assays.

The effect of exposure to Hg^2+^ on the growth of strain MSR33 was studied (Fig. 4). Cells were grown in LPTMS medium with succinate as the sole carbon source in the absence or presence of Hg^2+^ (0.04 mM). Hg^2+^ did not affect the growth of MSR33 (µ = 0.051 h^−1^), whereas the growth of strain CH34 decreased slightly in presence of Hg^2+^ (µ = 0.044 h^−1^) (Fig. 4). Strains MSR33 and CH34 showed a similar growth rate in absence of Hg^2+^. In addition, the effect of Hg^2+^ (0.04 mM) exposure at exponential phase of growth was studied. Interestingly, strain MSR33 growth was not affected after addition of Hg^2+^ at exponential phase (Fig. 4). In contrast, no further growth of strain CH34 was observed when 0.04 mM of Hg^2+^ were added at exponential phase.

**Figure 5:**
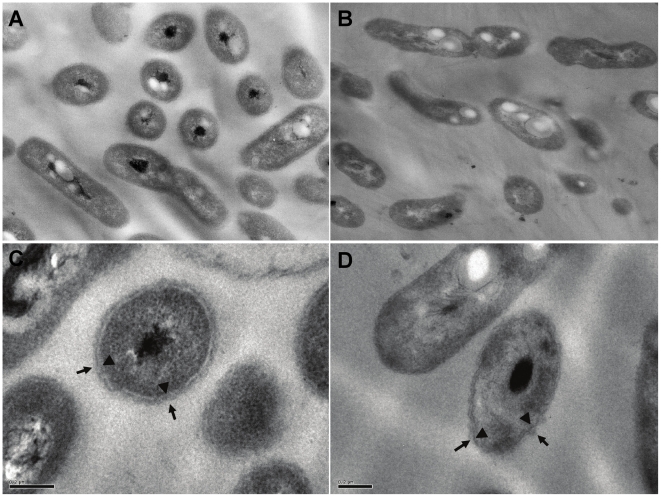
Ultrathin section of *C. metallidurans* strain MSR33 exposed to Hg^2+^. Cells grown in LPTMS medium with succinate until exponential phase were further incubated in presence of Hg^2+^ (0.04 mM) for 1 h. **A** and **C**, MSR33 cells; **B** and **D**, CH34 cells. Arrows and arrowheads indicate the outer and the inner membrane, respectively. The bars represent 0.2 µm.

To evaluate changes in cell morphology by exposure to mercury, cells of MSR33 grown on LPTMS medium with succinate as sole carbon source and exposed to Hg^2+^ were analyzed by transmission electron microscopy (Fig. 5). Strain MSR33 cells exposed to Hg^2+^ showed no changes in cell morphology. In contrast, CH34 cells exposed to Hg^2+^ exhibited a fuzzy outer membrane. Electron dense granules in the cytoplasm of MSR33 and CH34 strains exposed to Hg^2+^ were also observed (Fig. 5).

### Bioremediation of mercury-polluted water

**Figure 6:**
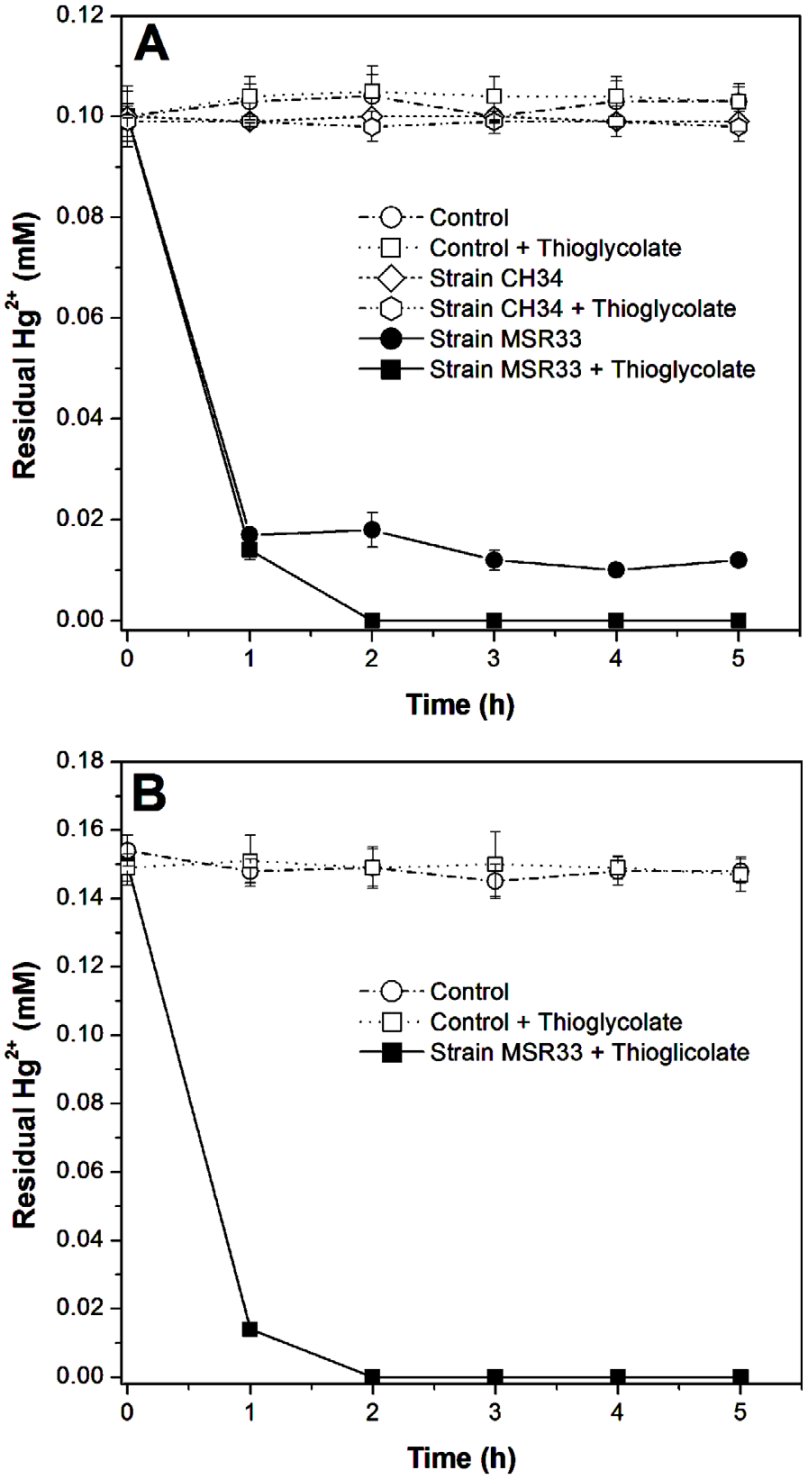
Effect of bioaugmentation with *C. metallidurans* strain MSR33 in Hg^2+^-polluted waters. **A**, removal of Hg^2+^ from water polluted with Hg^2+^ (0.10 mM) by strain MSR33 or strain CH34 in presence or absence of thioglycolate (5 mM). **B**, removal by strain MSR33 of Hg^2+^ from water polluted with Hg^2+^ (0.15 mM) in presence of thioglycolate (5 mM). Control assays without cells were incubated in presence or absence of thioglycolate (5 mM).

In this study, the effectiveness of *C. metallidurans* strain MSR33 as a biocatalyst for the bioremediation of two Hg^2+^-polluted aqueous solutions in a bioreactor was assessed. Bioaugmentation was studied in aqueous solutions containing Hg^2+^ (0.10 mM and 0.15 mM). The effect of addition of thioglycolate, a compound that provides an excess of exogenous thiol groups [41], [42] was evaluated on mercury bioremediation by strain MSR33. The effect of bioaugmentation by strain MSR33 on Hg^2+^ removal in two polluted waters is shown in Figure 6. In presence of thioglycolate, *C. metallidurans* strain MSR33 completely removed mercury (0.10 mM and 0.15 mM) from the two polluted waters after 2 h. In absence of thioglycolate, strain MSR33 removed 88% of Hg^2+^ (0.10 mM) after 3 h. In contrast, wild type *C. metallidurans* strain CH34 was not capable to remove mercury either in presence or absence of thioglycolate. No mercury removal was observed in the control bioreactors.

## Discussion

Mercury-polluted sites often contain other heavy metals. Therefore, for the bioremediation of mercury-polluted sites broad range heavy metal-resistant bacteria are required. *C. metallidurans* strain CH34 is a model heavy metal-resistant bacterium [27], [28]. The main goal of this work was to generate a heavy metal-resistant bacterial strain with resistance to inorganic and organic mercury species. Therefore, the heavy metal-resistant strain *C. metallidurans* CH34 with low resistance to mercury (II) and sensitive to organic mercury was modified by introducing the plasmid pTP6 that has been directly isolated from the environment and that encodes Mer proteins that confer a broad-range mercury resistance [26]. The transconjugant *C. metallidurans* strain MSR33 was characterized and applied for remediation of mercury-polluted waters.

In the present study, improved mercury resistance of the transconjugant strain MSR33 was obtained by incorporation of broad-host range plasmid pTP6 with a complex *mer* system as sole accessory element into *C. metallidurans* CH34. To our knowledge, this is the first study to use an IncP-1β plasmid directly isolated from the environment to generate a novel bacterial strain with improved mercury resistance. Therefore, and in contrast to genetically engineered bacteria, environmental application of transconjugant strain MSR33 is not subjected to biosafety regulation, which is an important advantage for bioremediation of mercury-polluted sites. This study showed that pTP6 stably replicated even without selective pressure as other IncP-1 plasmids that are tightly controlled by *korA* and *korB* genes [26]. Interestingly, IncP-1 plasmids have an extremely broad-host range in Gram-negative bacteria and are efficiently transferred from the host to soil bacteria [43], [44]. The natural plasmid pTP6 provided novel *merB* and *merG* genes and conferred a broad-spectrum mercury resistance to *C. metallidurans* strain MSR33. The presence of plasmid pTP6 in strain MSR33 provided also additional *mer* gene copies (*merR*, *merT*, *merP*, *merA*, *merD* and *merE* genes) to the host strain, improving also inorganic mercury resistance. The genetic redundancy may increase the robustness of organisms to mercury stress [45]. The MerA protein encoded by plasmid pTP6 belonged to different subclasses than MerA proteins of pMOL28 and pMOL30. On the other hand, plasmid pTP6 provides two copies of *merB* genes encoding MerB1 and MerB2 enzymes.

The mechanisms for mercury detoxification in strain MSR33 are shown in Figure 1. The expression of *mer* genes of pTP6 in strain MSR33 support growth of strain MSR33 with Hg^2+^ at high concentrations and in presence of CH_3_Hg^+^. Strain MSR33 possesses a high organic mercury resistance (CH_3_Hg^+^, 0.08 mM) and a high inorganic mercury resistance (Hg^2+^, 0.12 mM) (Table 1). The novel resistance to methylmercury is conferred by the organomercurial lyase MerB. The organomercurial lyase catalyzes the protonolysis of the carbon mercury bond, removing the organic ligand and releasing Hg^2+^. Inorganic mercury is 100 times less toxic than the CH_3_Hg^+^ form [9], [24]. An additional MerA enzyme improved strain MSR33 detoxification of Hg^2+^, and additional MerT and MerP proteins in strain MSR33 probably allow a rapid uptake of mercury avoiding an extracellular accumulation. The mercury resistance of strain MSR33 is higher than that of other recombinant strains such as *Pseudomonas putida* F1::*mer* [46]. The addition of *merTPAB* genes in *P. putida* F1 increased broad-spectrum mercury resistance in the range of 14–86%.

Strain MSR33 showed a similar growth rate in absence and presence of Hg^2+^ (Fig. 4). As expected, the presence of Hg^2+^ decreased the growth rate of strain CH34. Noteworthy, the growth of MSR33 was not affected by the addition of mercury (II) at exponential phase (Fig. 4C). On the other hand, an immediate cessation of growth of strain CH34 was observed after the addition of Hg^2+^ at exponential phase. The additional *mer* genes incorporated into strain MSR33 probably improve the mercury detoxification system. The effects of Hg^2+^ on growth are in accordance with the effects on cell morphology (Fig. 5). Cell membrane of strain MSR33 was not affected by the presence of mercury. In contrast, after the exposure to Hg^2+^ strain CH34 showed a fuzzy outer membrane. *Enterobacter* exposed to mercury also showed a fuzzy cell membrane [47]. After exposure to mercury, both strains MSR33 and CH34 contained electron-dense granules in the cytoplasm that probably are polyhydroxybutyrate. *C. metallidurans* CH34 harbors the genes for polyhydroxybutyrate synthesis [48] and probably accumulates these intracellular storage granules during stress.

The bioremediation potential of strain MSR33 of mercury-polluted aqueous solutions was evaluated in a bioreactor. Strain MSR33 completely removed Hg^2+^ (0.10 mM and 0.15 mM) from polluted aqueous solutions. The presence of thioglycolate was required for complete mercury removal in water. The presence of thiol molecules ensures that Hg^2+^ will be present as the dimercaptide, RS-Hg-SR. The dimercaptide form of Hg^2+^ is the substrate of the NADPH-depending mercuric reductase MerA [42]. Thiol compounds such as 2-mercaptoethanol, cysteine and thioglycolate improved mercury volatilization by *Escherichia coli*, *Pseudomonas* sp. strain K-62, bacterial strain M-1 and *Pseudomonas putida* strain PpY101 (pSR134) [21], [41], [49]–[53].

In the present study, strain MSR33 showed a high mercury volatilization rate, 6.8×10^−3^ ng Hg^2+^ cell^−1^ h^−1^. Saouter et al. [54] studied mercury removal from freshwater pond by resting cells of mercury-resistant *Aeromonas hydrophila* strain KT20 and *Pseudomonas aeruginosa* PAO1 derivative containing a plasmid carrying *mer* genes. The mercury volatilization rates of *A. hydrophila* KT20 and *P. aeruginosa* PAO1 derivative were 1.0×10^−3^ ng Hg^2+^ cell^−1^ h^−1^ and 2.4×10^−4^ ng Hg^2+^ cell^−1^ h^−1^, respectively. Okino et al. [53] studied mercury removal from a mercury-polluted aqueous solution with the genetically engineered bacterium, *P. putida* strain PpY101 (pSR134) showing a volatilization rate of 6.7×10^−3^ ng Hg^2+^ cell^−1^ h^−1^.

*C. metallidurans* MSR33 is a stable transconjugant strain carrying plasmid pTP6, which conferred a novel organomercurial resistance and improved significantly the resistance to Hg^2+^. Strain MSR33 was able to cleave the organic moiety from methylmercury and to reduce Hg^2+^ into a less toxic Hg elemental form. Strain MSR33 efficiently removed Hg^2+^ (0.10 mM and 0.15 mM) through mercury volatilization from mercury-contaminated waters. This study suggests that strain MSR33 may be useful for mercury bioremediation of contaminated water bodies and industrial wastewater. In addition, strain MSR33 is an interesting biocatalyst for bioremediation of mercury-polluted soils.
